# Dapagliflozin attenuates progression from prefrailty to frailty in patients with hypertension and chronic kidney disease

**DOI:** 10.1172/JCI205416

**Published:** 2026-11-02

**Authors:** Pasquale Mone, Davide Viggiano, Antonella Pansini, Maria Virginia Soldovieri, Giada Sabatelli, Valeria Visco, Gaetano Macina, Eugenio Boccalone, Michele Ciccarelli, Giuseppe Martinelli, Marco Di Mauro, Carmine Vecchione, Maria Morgante, Fahimeh Varzideh, Germano Guerra, Gaetano Santulli

**Affiliations:** 1 Department of Medicine and Health Sciences, University of Molise, Campobasso, Italy.; 2 Clinica Montevergine, GVM Care and Research, Mercogliano (Avellino), Italy.; 3 Division of Internal Medicine, Veneziale Hospital, Isernia, Italy.; 4 Department of Translational Medical Sciences, University of Campania “Luigi Vanvitelli”, Naples, Italy.; 5 Local Health Unit of the Italian Ministry of Health (ASL), Avellino, Italy.; 6 Department of Medicine, Surgery, and Dentistry, “Scuola Medica Salernitana”, University of Salerno, Baronissi (Salerno), Italy.; 7 Local Health Unit of the Italian Ministry of Health (ASL), Naples, Italy.; 8 Vascular Physiopathology Unit, IRCCS “Neuromed - Mediterranean Neurological Institute”, Pozzilli, Italy.; 9 San Pio Hospital, Benevento, Italy.; 10 Department of Molecular, Cellular, and Biomedical Sciences, City University of New York, School of Medicine, Manhattan, New York, USA

**Keywords:** Aging, Cardiology, Clinical Research, Cardiovascular disease, Heart failure, Hypertension

## Abstract

**BACKGROUND:**

Chronic kidney disease (CKD) and hypertension share common pathophysiological pathways contributing to vascular damage, endothelial dysfunction, and physical decline, particularly in older adults with prefrailty. To evaluate the relationship among renal injury, physical performance, and frailty progression in hypertensive older adults with CKD and assess whether dapagliflozin associates with reduced frailty progression, we designed the study DAPA-FORTRESS (DAPAgliflozin and Frailty prOgression in hypeRTension and chRonic kidnEy diSeaSe).

**METHODS:**

We enrolled 135 prefrail hypertensive patients with CKD aged >65 years and followed them for 1 year. After coarsened exact matching, our balanced cohort had 105 patients (52 dapagliflozin). Associations between albuminuria, estimated glomerular filtration rate (eGFR), and physical performance were analyzed using correlation and regression models. Effect of dapagliflozin on frailty progression was evaluated using multivariable logistic regression.

**RESULTS:**

A significant inverse association between gait speed and albuminuria (*r*^2^ = 0.48, *P* < 0.0001) and significant positive linear association between gait speed and eGFR (*r*^2^ = 0.18; *P* < 0.0001) were observed. Patients with eGFR <60 mL/min/1.73 m^2^ exhibited significantly worse physical performance than those with higher eGFR (*P* = 0.0003). In adjusted analyses, receiving dapagliflozin was independently associated with lower odds of progressing to frailty at 1 year (OR = 0.130; CI 0.045–0.381; *P* < 0.001).

**CONCLUSION:**

In older hypertensive patients with CKD, greater renal injury is associated with worse physical performance, while dapagliflozin use reduces the risk of frailty progression. These findings indicate a potential protective role of sodium–glucose cotransporter 2 inhibition in mitigating functional decline in this high-risk population.

**FUNDING:**

NIH: National Heart, Lung, and Blood Institute (R01-HL146691), National Institute of Diabetes and Digestive and Kidney Diseases (R01-DK123259); American Heart Association (AHA- 24IPA1268813, AHA-22-915561, AHA-24-1195524).

## Abstract 2

In older adults with CKD and hypertension, worse kidney health is linked to poorer physical function, and dapagliflozin may help prevent frailty progression.

## Introduction

Chronic kidney disease (CKD) is a common complication of hypertension, with both conditions sharing similar pathophysiological mechanisms (1, 2). Prefrailty is a well-established condition that contributes to the development of adverse events related to CKD and hypertension, particularly in older adults (3–5). Comorbid conditions, such as hypertension and CKD, are frequently associated with organ damage, which may promote the development of prefrailty, frailty, and functional decline (6, 7). Albuminuria is a marker of kidney damage and an important predictor of CKD and cardiovascular outcomes (8, 9). Dapagliflozin, a sodium–glucose cotransporter 2 (SGLT2) inhibitor, has been shown to provide benefits beyond glycemic control, including a reduced future risk of dialysis initiation (10–13). In addition, SGLT2 inhibitors have become widely prescribed not only for their established benefits in diabetes, CKD, and heart failure, but also for their expanding spectrum of pleiotropic effects across multiple cardiometabolic and systemic disorders (14–18).

The landmark SGLT2 inhibitor trials systematically underrepresented the elderly patients who constitute the majority of the real-world CKD population; trial-eligible patients with CKD in routine practice have been shown to be older than the participants enrolled in these trials and carried a higher burden of cardiovascular disease (19, 20). Therefore, the evidence supporting SGLT2 inhibitor use in CKD is derived predominantly from younger patients with fewer comorbidities.

In this context, we hypothesized that greater renal and vascular injury would be associated with a worse physical performance and a higher burden of prefrailty in older hypertensive patients with CKD. Furthermore, we posited that treatment with dapagliflozin would be associated with a reduced progression of frailty, potentially through attenuation of systemic vascular and renal injury. To test these hypotheses, we designed an observational prospective study named DAPA-FORTRESS (DAPAgliflozin and Frailty prOgression in hypeRTension and chRonic kidnEy diSeaSe).

## Results

A total of 196 prefrail older adults with hypertension and CKD were screened for study participation. Among these individuals, 38 did not meet the predefined inclusion criteria, and an additional 23 declined to participate. Consequently, 135 patients were enrolled and successfully completed the study (Figure 1); all patients in the dapagliflozin group received the treatment for the entire year of observation. Baseline clinical characteristics of the entire population participating in the DAPA-FORTRESS study are summarized in Supplemental Table 1; supplemental material available online with this article; https://doi.org/10.1172/JCI205416DS1 The decision to prescribe dapagliflozin was driven by clinicians’ assessment of each patient’s clinical profile, including age, renal function, and diabetes. These factors could influence frailty progression, creating potential confounding elements. Therefore, coarsened exact matching (CEM) was applied to restructure the cohort into strata balanced on key baseline covariates (age, sex, estimated glomerular filtration rate [eGFR], and diabetes), ensuring that the dapagliflozin and no-dapagliflozin groups were comparable before the outcome analysis and that any residual difference in 1-year frailty could be more confidently attributed to dapagliflozin exposure rather than to the patient characteristics that drove the prescribing decision. After CEM, 105 patients (52 in the dapagliflozin group and 53 in the no-dapagliflozin group) were analyzed (Table 1). In a simple linear regression analysis, a significant inverse association between gait speed and albuminuria was observed (*r*^2^: 0.48; *r* = –0.691; *P* < 0.0001; Figure 2A). In line with this observation, we also detected a significant positive linear association between gait speed and eGFR (*r*^2^ = 0.18; *r* = 0.424; *P* < 0.0001; Figure 2B). However, these relationships should not be interpreted as causal: both renal injury and impaired physical performance may share common underlying drivers (including vascular disease, chronic inflammation, diabetes, and accelerated aging) that independently contribute to both findings. The observed associations therefore reflect a correlation between renal and physical function markers rather than evidence that renal involvement directly causes gait impairment. To further investigate the contribution of kidney function to physical impairment, participants were then stratified according to eGFR categories. This analysis demonstrated significantly worse physical performance among patients with eGFR <60 mL/min/1.73 m^2^ compared with those with eGFR ≥60 mL/min/1.73 m^2^ (*P* = 0.0003; Figure 3).

Having established that renal function markers are associated with physical performance, we next examined whether dapagliflozin treatment was independently associated with frailty outcomes after accounting for potential confounders. After calculating univariate estimates (Supplemental Table 2), a multivariable logistic regression model was constructed with progression to frailty at 1 year as the binary outcome (Table 2). In this model, dapagliflozin use was independently associated with lower odds of progressing to frailty at 1 year (OR = 0.130, CI 0.045–0.381; *P* < 0.001) after adjustment for age, eGFR, gait speed, and diabetes. The treatment effect observed in the regression model is also depicted in Figure 4, which shows the distribution of frailty states at 1-year follow-up by treatment group. Patients receiving dapagliflozin were less likely to transition from prefrailty to frailty compared with untreated patients.

## Discussion

The present study, by examining dapagliflozin’s association with frailty outcomes in an exclusively elderly population with CKD and hypertension, addresses a population that is both at highest risk of adverse outcomes and most underrepresented in the existing evidence base. Focusing on an elderly cohort is particularly important if we consider that the benefit-risk profile in the very old (particularly those with frailty) remains poorly characterized (21, 22). This gap is consequential because frailty affects approximately one-third of patients with CKD and predicts mortality and cardiovascular hospitalization even after adjustment for age and kidney function (23, 24).

In line with this framework, our findings support the concept that established markers of organ damage, including albuminuria and reduced eGFR, should not be interpreted solely as renal endpoints, but rather as integrated indicators of systemic vascular injury and global endothelial dysfunction that adversely impact physical performance (24–29). Our results suggest that renal biomarkers may serve as particularly powerful indicators of functional reserve in elderly patients. Mechanistically, hypertension drives endothelial dysfunction and target-organ damage, increasing the risk of nephropathy and promoting maladaptive vascular remodeling characterized by chronic inflammation, oxidative stress, and accelerated atherosclerosis (6, 30–32). The coexistence of hypertension and CKD may exert synergistic effects, amplifying physical impairment in prefrail older adults and accelerating the transition toward overt frailty (1, 8). Endothelial dysfunction and microvascular impairment may contribute to reduced skeletal muscle perfusion and mitochondrial dysfunction, thereby directly influencing physical performance and gait speed (33–35).

Our results are compatible with previously proposed mechanisms involving vascular, endothelial, and renal effects of SGLT2 inhibition (36), though these mechanisms were not directly assessed in the present study. The consistency of this benefit across cardiovascular and renal outcomes (37, 38), including prespecified frailty subgroup analyses (39, 40) and superaged populations (41), supports a mechanism extending beyond glucose lowering. The consistency and magnitude of the observed associations are clinically meaningful and complements the prespecified frailty subgroup analyses of the DELIVER and DAPA-CKD trials, which demonstrated that dapagliflozin’s efficacy and safety were maintained across the frailty spectrum (39, 40, 42). Nevertheless, vigilant monitoring for volume depletion, hypotension, and genitourinary infections remains warranted in very old patients with CKD (42–44); thus, a personalized and mechanism-oriented therapeutic approach that simultaneously targets blood pressure control, endothelial dysfunction, and downstream vascular complications might be particularly effective in mitigating age-related physical decline (41, 45–49).

Several limitations should be acknowledged. The relatively homogeneous inclusion and exclusion criteria of the DAPA-FORTRESS study may improve clinical comparability but limit generalizability to younger individuals, those with more advanced CKD, or patients with secondary hypertension. CEM ensures balance on observed covariates, but the exclusion of patients lacking common support across treatment strata may have systematically removed individuals with more severe disease, potentially restricting the estimated treatment effect to a less advanced CKD population. Furthermore, CEM balances only measured confounders; unmeasured factors influencing both the prescribing decision and frailty outcomes (physician preference, patient adherence, nutritional status, social support) remain potential sources of residual confounding. Finally, the high transition rate in the nondapagliflozin group should be interpreted cautiously: while it likely reflects the substantial comorbidity burden of this cohort, it exceeds rates reported in general community-dwelling populations and warrants validation in larger cohorts. Nevertheless, this study adds to the growing literature linking renal biomarkers to physical decline (50–52) by specifically examining this relationship within a prefrail hypertensive CKD population and by providing evidence that dapagliflozin use is associated with reduced frailty progression in an exclusively elderly cohort. Future investigations and randomized clinical trials based on larger multicenter cohorts with extended follow-up are warranted to validate our observations and to determine whether early, targeted strategies aimed at vascular and renal protection can effectively prevent frailty progression.

## Methods

### Sex as a biological variable.

We examined both female and male individuals.

### Study design.

The DAPA-FORTRESS study involves consecutive prefrail older patients with hypertension and CKD, recruited between March 2021 and September 2023 at the ASL of Avellino, Italy. Inclusion criteria were age >65 years, a prior confirmed diagnosis of hypertension in the absence of secondary causes, CKD, and prefrailty status (53). Patients were excluded if they did not meet these criteria, if they had cancer or stroke, or if they declined participation. The effects of dapagliflozin (10 mg/d) on frailty status were evaluated in both groups over a 1-year follow-up period.

### Physical frailty assessment.

Patients were examined at baseline and after 1-year follow-up. Frailty was defined according to Fried criteria (≥3 components), with prefrailty defined as 1–2 components among low physical activity, unintentional weight loss, exhaustion, weakness, and slowness, as described (22, 53). Gait speed, assessed using the 5-meter walking test (3), was used as the primary indicator of physical impairment.

### Statistics.

Data are presented as mean ± SD or percentages, as appropriate. To minimize baseline differences between patients treated and not treated with dapagliflozin and to reduce potential confounding inherent to the observational design, CEM was performed before the outcome analyses. Among the initially enrolled cohort of 135 patients (65 receiving dapagliflozin and 70 not receiving dapagliflozin), CEM was applied using 4 clinically relevant baseline variables selected a priori: age, eGFR, sex, and diabetes status. Before matching, age was coarsened into 5 categories (<70, 70–79, 80–89, 90–95, and >95 years), and eGFR into 3 categories (<20, 20–59, and ≥60 mL/min/1.73 m²), and sex and diabetes status were retained as binary variables (female/male and yes/no, respectively). Each patient was assigned to a unique stratum defined by the cross-classification of these 4 variables. Exact matching was performed within these coarsened strata, and a stratum was retained only if it contained at least 1 dapagliflozin-treated and at least 1 untreated patient. All patients within qualifying strata were retained rather than selecting individual one-to-one matched pairs, allowing unequal numbers of treated and untreated patients within a stratum while ensuring covariate overlap. Patients belonging to strata containing only 1 treatment group were excluded from the matched analytic population. This procedure yielded a CEM cohort of 105 patients (52 receiving dapagliflozin and 53 not receiving dapagliflozin), representing the region of covariate overlap defined by the prespecified matching criteria. CEM was preferred over propensity score matching because it guarantees covariate balance within clinically interpretable strata without estimating a propensity model, thereby reducing model dependence and the risk of mis-specification, whereas propensity score matching can paradoxically increase imbalance as the data become more balanced and relies on a model whose correct specification cannot be verified independently of the matching outcome itself (54, 55). This approach has been previously applied in nephrology cohorts, including in CKD populations (56, 57). The matched cohort was then used for all the subsequent analyses, including the association between dapagliflozin exposure and 1-year frailty outcomes. Continuous variables were compared using Welch’s *t* test and categorical variables using Fisher’s exact test. The relationships between gait speed and renal function parameters (albuminuria and eGFR) at baseline were evaluated using simple linear regression analyses. Least-squares regression lines were fitted, and the coefficient of determination was used to quantify the proportion of variance in gait speed explained by each predictor. We then compared physical performance according to eGFR categories (<60 vs. ≥60 mL/min/1.73 m^2^) (58–60). The association between dapagliflozin use and 1-year progression to frailty was assessed using multivariable logistic regression, with Wald statistics used to assess coefficient significance. Dapagliflozin was the primary exposure, and age, eGFR, gait speed, and diabetes status were selected a priori as covariates based on their established clinical relevance to treatment assignment and frailty risk and, where applicable, their potential role as confounding factors. Age and eGFR were modeled as continuous variables, gait speed as a continuous measure of physical function, and diabetes as a binary variable. Creatinine and albuminuria were not included in the multivariable model to avoid redundant adjustment for multiple measures of renal disease and to limit the potential for multicollinearity. Statistical analyses were performed using IBM SPSS Statistics version 26 (IBM Corp., Armonk, New York, USA); Python version 3.13.15 with NumPy 2.5.2, pandas 3.0.5, and Matplotlib 3.11.1 (Python Software Foundation, Wilmington, Delaware, USA); and GraphPad Prism version 10.2 (Dotmatics, Boston, Massachusetts, USA). A *P* value less than 0.05 was considered significant.

### Study approval.

All participants provided written informed consent prior to enrollment. The study was conducted in accordance with the principles outlined in the Declaration of Helsinki, and the study protocol received approval from the Ethics Committee of Campania Nord.

### Data availability.

Data supporting the figures are provided in the Supporting Data Values file. All data are available from the corresponding author upon reasonable request.

## Manuscript information

### Author contributions.

G Santulli conceived and designed the study, developed the methodology, performed the formal analysis together with FV, and supervised and administered the project. PM curated the data, contributed to the investigation, and wrote the original draft together with FV. FV also contributed to the investigation and to the formal analysis. AP contributed to the investigation and, together with G Santulli, reviewed and edited the manuscript. MVS, G Sabatelli, VV, EB, MC, G Martinelli, MDM, and MM contributed to the investigation. G Macina contributed to the investigation and performed validation together with DV. DV also carried out visualization together with CV and GG. GG additionally provided resources. All authors have read and agreed to the published version of the manuscript.

### AI disclosure.

The authors declare that no AI tools were used in the original research for or writing of this article.

### Conflict of interest.

The authors have declared that no conflict of interest exists.

### Funding.

This study was supported by the following:

- GS in part by the National Institutes of Health (NIH): National Heart, Lung, and Blood Institute (NHLBI: R01-HL146691, R01-HL164772, R01-HL159062, T32-HL144456, T32-HL172255), and National Institute of Diabetes and Digestive and Kidney Diseases (NIDDK: R01-DK123259, R01-DK033823) and by the American Heart Association (AHA: 24IPA1268813).
- FV in part by the American Heart Association (22POST915561 and 24POST1195524).

This work is the result of NIH funding, in whole or in part, and is subject to the NIH Public Access Policy. Through acceptance of this federal funding, the NIH has been given a right to make the work publicly available in PubMed Central.

## Supplementary Material

Supplemental data

ICMJE disclosure forms

Supporting data values

## Figures and Tables

**Figure 1:**
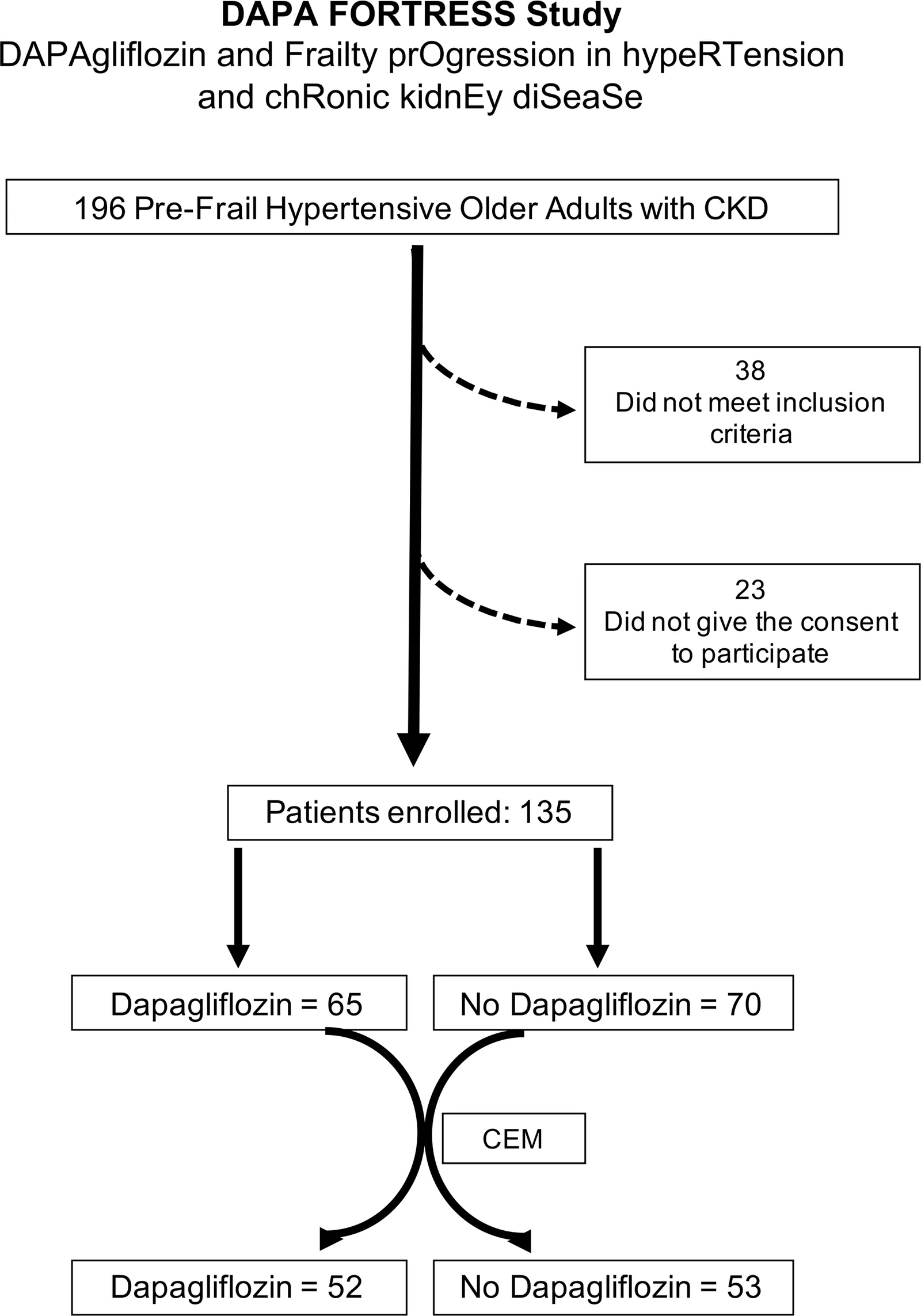
Flowchart of the study. CEM, coarsened exact matching; CKD, chronic kidney disease.

**Figure 2:**
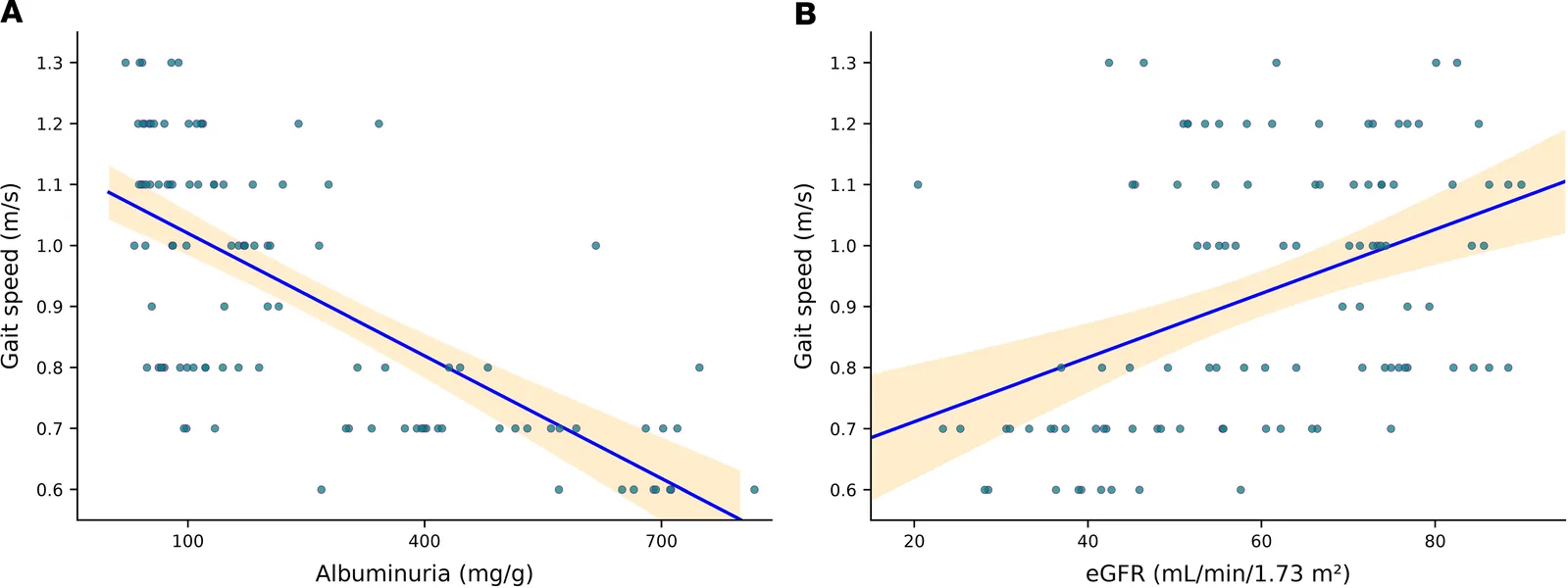
Gait speed is significantly associated with albuminuria and eGFR. Correlation between gait speed and albuminuria (**A**, *r*^2^: 0.48; *r* = –0.691; *P* < 0.0001) and between gait speed and eGFR (**B**, *r*^2^ = 0.18; *r* = 0.424; *P* < 0.0001) in a simple linear regression analysis. Data obtained at baseline (*n* = 105). The solid blue line represents the least-squares regression line; the shaded orange bands denote the 95% confidence interval around the fitted values.

**Figure 3:**
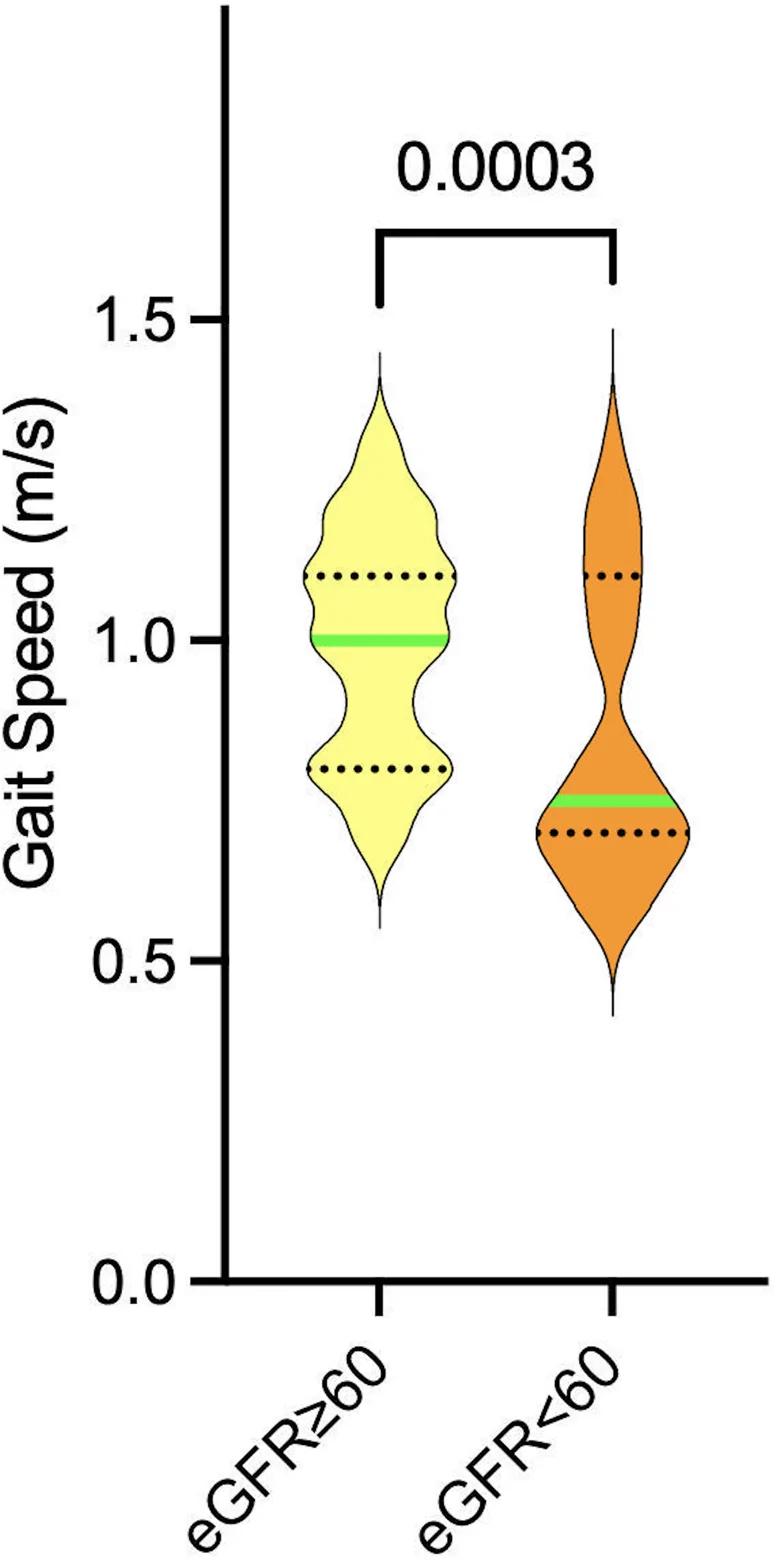
Gait speed according to eGFR category. Data obtained at baseline (*n* = 105: 51 in the group with eGFR ≥60 mL/min/1.73 m^2^ vs. 54 in the group with eGFR <60 mL/min/1.73 m^2^); *P* = 0.0003, Welch’s 2-sample *t* test. Within the violin plots, the green line represents the median, and the dotted lines represent the interquartile values.

**Figure 4:**
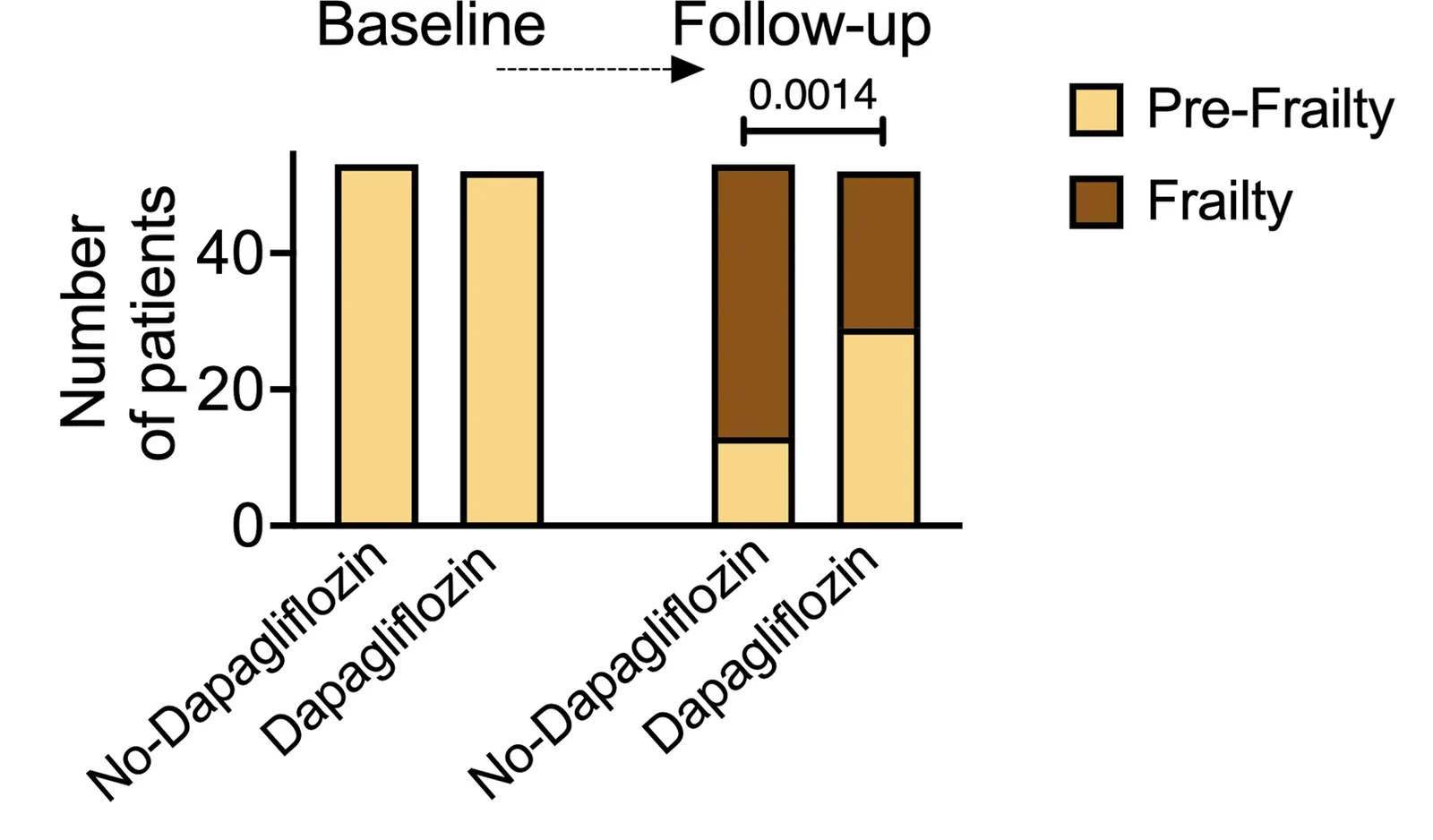
Dapagliflozin treatment reduces the transition from a prefrail to a frail status. Frailty transition at 1-year follow-up in patients treated or not with dapagliflozin (44.2% vs. 75.5%, respectively; *P* = 0.0014, Fisher’s exact test). Bars represent the proportion of patients in each frailty state at 1-year follow-up.

**Table 2:**
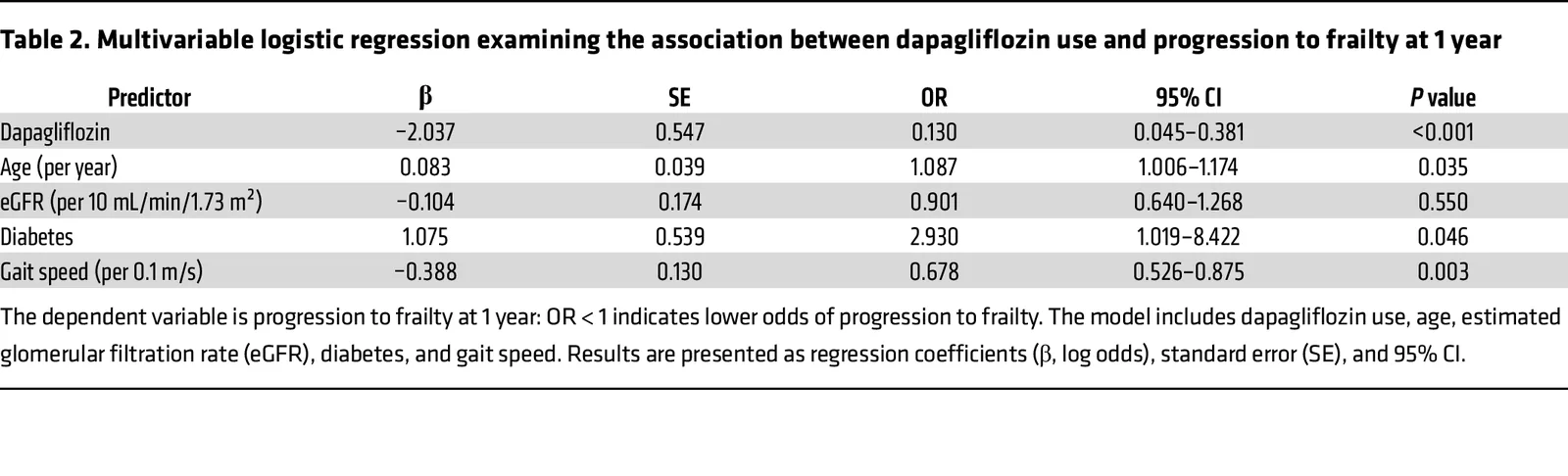
Multivariable logistic regression examining the association between dapagliflozin use and progression to frailty at 1 year

**Table 1:**
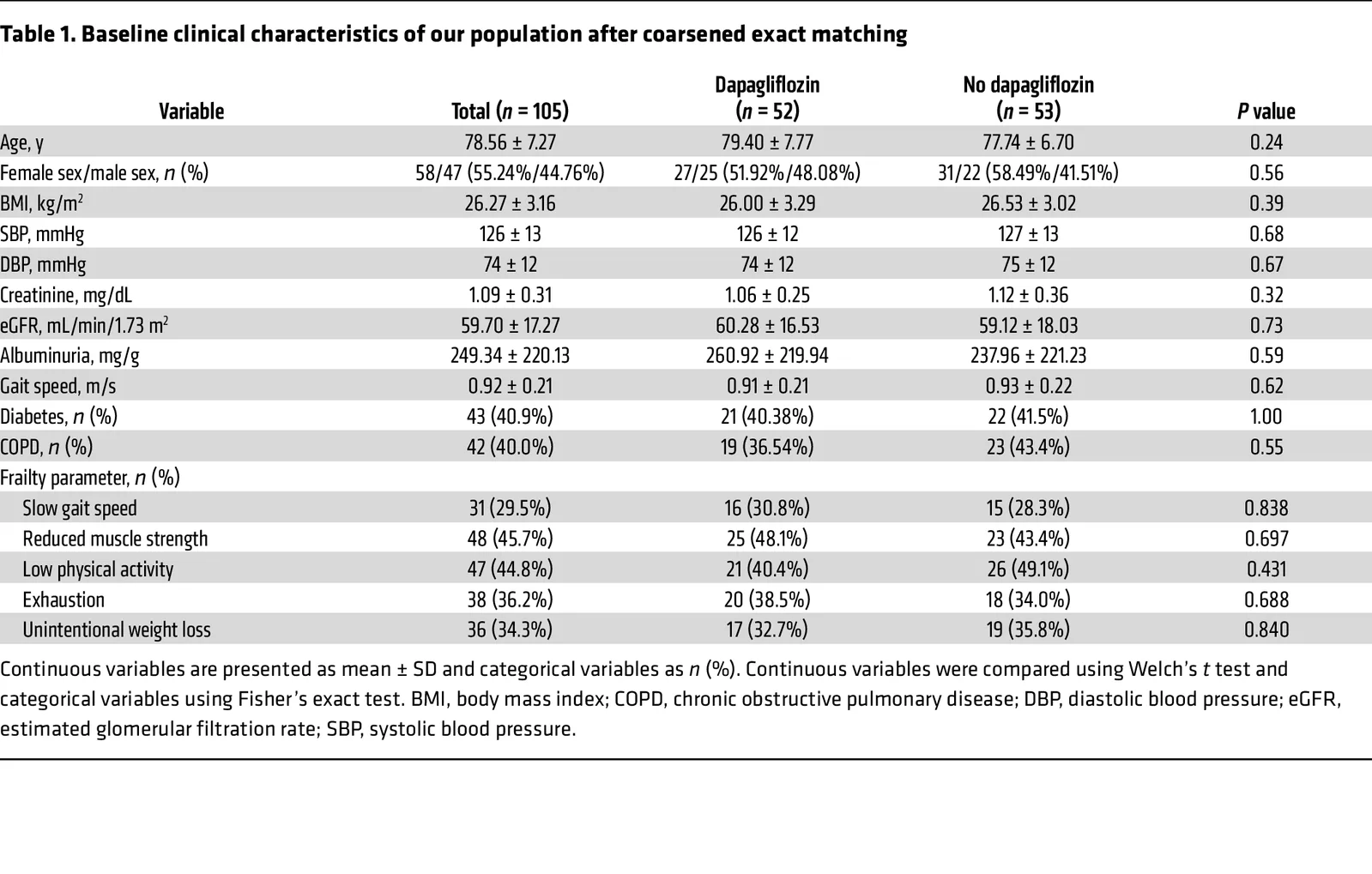
Baseline clinical characteristics of our population after coarsened exact matching

## Version 1 11/02/2026

Electronic publication
