# Supplementary material for: Dapagliflozin attenuates progression from prefrailty to frailty in patients with hypertension and chronic kidney disease
Source: J Clin Invest. 2026 Nov 2;136(21):e205416. doi: 10.1172/JCI205416 (PMC13722378; doi:10.1172/JCI205416)
Supplement: Supplementary file 1 [file jci-136-205416-s104.pdf]

Dapagliflozin attenuates progression from pre-frailty to frailty in patients with hypertension and chronic kidney disease

<u>Supplementary Material</u>

Variable	Total (n = 135)	Dapagliflozin (n = 65)	No Dapagliflozin (n = 70)	*P* value
**Age, years**	80.61 ± 8.85	80.54 ± 9.70	80.69 ± 8.04	0.92
**Female sex, n (%)**	73 (54.07%)	34 (52.31%)	39 (55.71%)	0.73
**BMI, kg/m²**	25.87 ± 2.97	25.74 ± 3.06	26.00 ± 2.90	0.61
**SBP, mmHg**	132 ± 15	130 ± 14	133 ± 15	0.31
**DBP, mmHg**	77 ± 12	76 ± 12	78 ± 12	0.48
**Creatinine, mg/dL**	1.31 ± 0.51	1.26 ± 0.48	1.36 ± 0.54	0.27
**eGFR, mL/min/1.73 m²**	52.68 ± 21.18	56.81 ± 16.71	48.84 ± 24.11	0.03
**Albuminuria, mg/g**	357 ± 288	333 ± 250	379 ± 320	0.35
**Gait speed, m/s**	0.87 ± 0.21	0.87 ± 0.21	0.87 ± 0.22	0.89
**Diabetes, n (%)**	57 (42.22%)	34 (52.31%)	23 (32.86%)	0.02
**COPD, n (%)**	59 (43.70%)	32 (49.23%)	27 (38.57%)	0.23
**Frailty parameter, n (%)**				
**Slow gait speed**	44 (32.6%)	18 (27.7%)	26 (37.1%)	0.27
**Reduced muscle strength**	63 (46.7%)	28 (43.1%)	35 (50.0%)	0.49
**Low physical activity**	58 (43.0%)	24 (36.9%)	34 (48.6%)	0.22
**Exhaustion**	47 (34.8%)	20 (30.8%)	27 (38.6%)	0.37
**Unintentional weight loss**	41 (30.4%)	16 (24.6%)	25 (35.7%)	0.19

Supplementary Table 1: Baseline clinical characteristics of our population before coarsened exact matching.

Continuous variables are presented as mean ± SD and categorical variables as n (%). Continuous variables were compared using Welch's t-test, and categorical variables using Fisher's exact test. BMI: body mass index; COPD: chronic obstructive pulmonary disease; DBP: diastolic blood pressure; eGFR: estimated glomerular filtration rate; SBP: systolic blood pressure.

Variable	β (log-odds)	SE(β)	OR	95% CI	*P* value
**Dapagliflozin, Yes *vs* No**	−1.356	0.424	0.258	0.112–0.592	0.0014
**Age, per year**	0.082	0.030	1.086	1.024–1.151	0.0059
**Sex, Female *vs* Male**	−0.620	0.409	0.538	0.241–1.200	0.1298
**BMI, per kg/m²**	0.009	0.063	1.009	0.891–1.143	0.8890
**SBP, per 10 mmHg**	−0.061	0.154	0.941	0.696–1.271	0.6922
**DBP, per 10 mmHg**	−0.024	0.161	0.977	0.712–1.338	0.8825
**Creatinine, per 0.1 mg/dL**	0.319	0.098	1.376	1.134–1.668	0.0012
**eGFR, per 10 mL/min/1.73 m²**	−0.359	0.129	0.698	0.542–0.899	0.0053
**Albuminuria, per 100 mg/g**	0.452	0.131	1.572	1.216–2.032	0.0006
**Gait speed, per 0.1 m/s**	−0.399	0.107	0.671	0.544–0.828	0.0002
**Diabetes, Yes *vs* No**	0.885	0.424	2.422	1.054–5.565	0.0372
**COPD, Yes *vs* No**	0.470	0.414	1.600	0.711–3.602	0.2563

Supplementary Table 2. Unadjusted associations between candidate predictors and frailty progression at 1 year, estimated using univariable logistic regression models.

Each variable was entered individually (unadjusted) into a logistic regression model, with progression to frailty at 1 year (1 = progressed, 0 = did not progress) as the dependent variable; n=105. An OR (odds ratio) < 1 indicates lower odds of frailty progression. SE = standard error of β; 95% CI = 95% confidence interval for the OR.
